# Regulation of anti-tumour effects of Paris polyphylla saponins via ROS: molecular mechanisms and therapeutic potentials

**DOI:** 10.3389/fphar.2025.1611911

**Published:** 2025-07-02

**Authors:** Jia Liu, Yongping Mu, Ke Qi, Jiayi Li, Yang Hu

**Affiliations:** ^1^ Inner Mongolia Medical University, Hohhot, Inner Mongolia, China; ^2^ Department of Clinical Test Laboratory, Hohhot First Hospital, Hohhot, Inner Mongolia, China

**Keywords:** Paris polyphylla, reactive oxygen species, anti-cancer, mechanism, saponins

## Abstract

Reactive oxygen species (ROS) exhibit a dual regulatory role in cancer biology. While moderate ROS levels promote tumorigenesis via DNA mutagenesis, excessive ROS accumulation induces cancer cell death through oxidative stress. Therefore, ROS homeostasis represents a promising therapeutic target in oncology. Collectively, ROS exhibit context-dependent and multifaceted roles in cancer progression. Emerging evidence highlights the anticancer potential of traditional Chinese medicine (TCM), particularly *Paris polyphylla* saponin (PPS). PPS modulates oxidative stress through precision targeting of ROS-associated signaling pathways, thereby inducing apoptosis, cell cycle arrest, autophagy, and ferroptosis. These mechanisms collectively suppress tumor growth, metastasis, and angiogenesis, while concurrently mitigating inflammatory responses. Notably, PPS potentiates the efficacy of chemotherapeutic agents by reversing multidrug resistance in refractory cancer cells. The bioactive constituents of PPS, polyphyllin and polyphyllinositol, exhibit potent antitumor activity in preclinical models. This study systematically elucidates the molecular mechanisms underlying PPS-mediated anticancer effects via ROS targeting, offering a robust theoretical framework and translational insights for future oncology research.

## 1 Introduction

Malignant tumors represent a critical global public health challenge, significantly compromising human health (Cronin et al., 2022; Miller et al., 2022). Cancer incidence and mortality rates are steadily escalating, driven by population aging and cumulative environmental risk exposure (Liao et al., 2019; Qin et al., 2018; Sparg et al., 2004; Torre et al., 2016). Globally, an estimated 19 million incident malignancies and 10 million attributable mortality were encompassed in 2020 (Sung et al., 2021), with projections indicating a surge to over 30 million new cases by 2040 (Murthy et al., 2024). Although conventional therapies (e.g., surgical resection, radiotherapy, and chemotherapy) have markedly improved patient survival, their clinical utility is constrained by dose-limiting toxicities, acquired drug resistance, and limited efficacy against metastatic tumors (Qin et al., 2018; Torre et al., 2016).

Natural medicines are now regarded as promising candidates in oncology research due to their multifaceted therapeutic potential. Multi-target strategies involving bioactive compounds from traditional Chinese medicine (TCM) are gaining prominence for their dual capacity to augment therapeutic outcomes while mitigating toxicity and overcoming chemoresistance (Xiang et al., 2019; Zhang et al., 2021). Among TCM-derived anticancer agents, *Paris polyphylla* saponins (PPS)—the core bioactive constituents of *P. polyphylla*—exhibit potent antitumor activity against multiple malignancies, including lung (Kong et al., 2010), gastric, and colorectal carcinomas (Teng et al., 2015). Notably, PPS modulate the redox equilibrium of reactive oxygen species (ROS), triggering tumor cell cycle arrest, apoptotic and autophagic cell death, while concurrently suppressing angiogenesis and metastatic dissemination. This ROS-dependent antitumor mechanism offers a novel paradigm for designing precision-targeted anticancer therapeutics. This review synthesizes recent advances in PPS-mediated ROS signaling modulation, systematically dissecting its molecular underpinnings and evaluating its translational prospects for anticancer drug development.

This research seeks to systematically explore the antitumor mechanisms of PPS through ROS level modulation, with the goal of proposing novel therapeutic strategies for cancer management. By elucidating the molecular mechanisms, signaling networks, and translational potential of PPS-mediated ROS generation, this research seeks to establish a foundational framework for developing precision-targeted antitumor agents and advancing their clinical applicability.

## 2 Chemical composition and bioactive properties of PPS

The genus *Paris* (Liliaceae family) comprises 24 species, of which 22 are endemic to China. *Paris polyphylla* is the most medicinally significant species within this genus, owing to its abundant germplasm diversity and phytochemical richness. To date, over 320 distinct chemical constituents have been identified in *P. polyphylla*, encompassing steroidal saponins (Zhou et al., 2021), C-21 steroids, phytosterols, ecdysteroids, pentacyclic triterpenoids, and flavonoids (Liu Y. et al., 2023). Of these,the steroidal saponins—collectively termed PPS—represent the predominant bioactive constituent (Ding et al., 2021).

**FIGURE 1:**
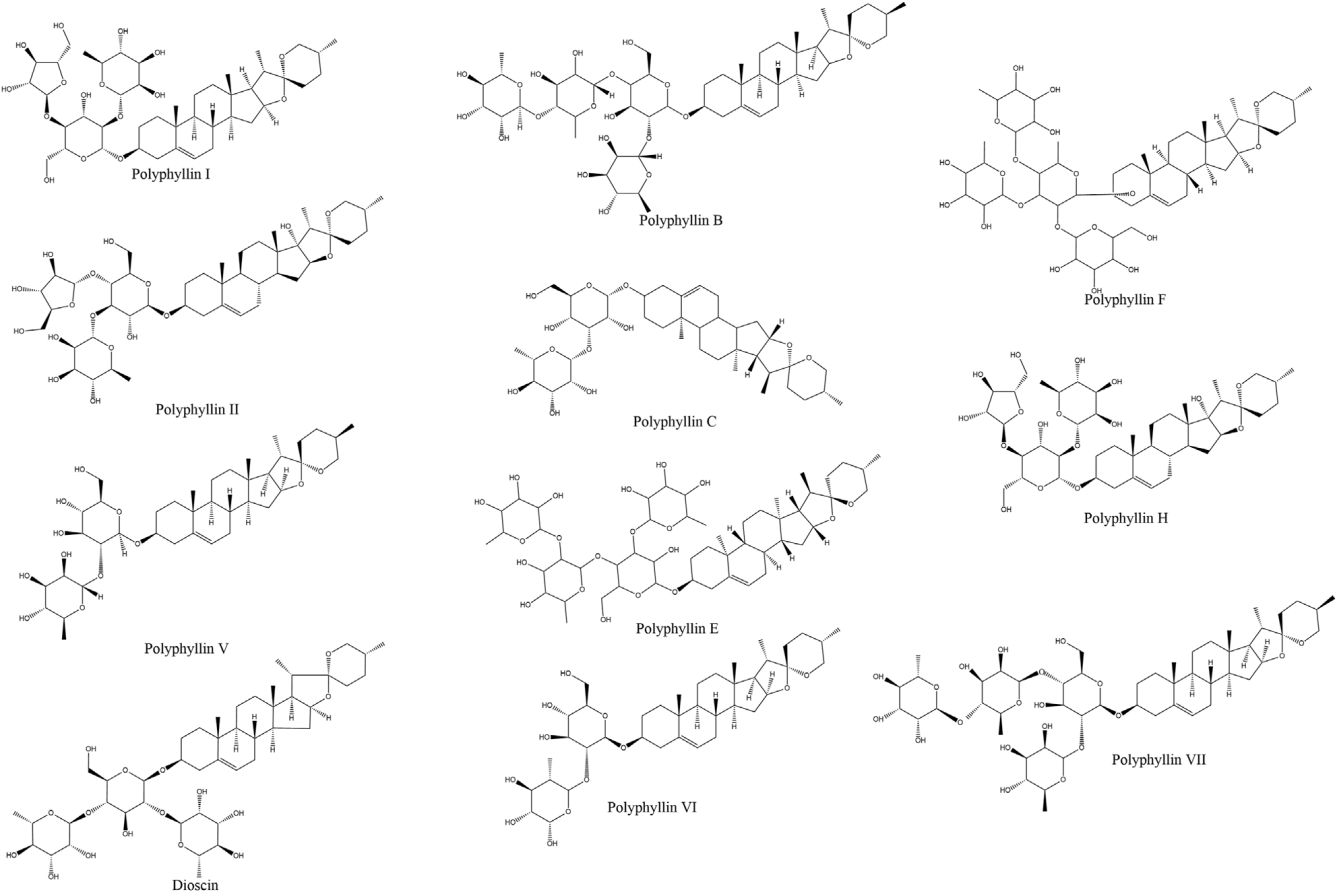
Rhizoma Paridis and its anti-cancer bioactive ingredients Rhizoma Paridis saponins (RPS). Commercially available RPS include polyphyllin I/polyphyllin D, polyphyllin II, Dioscin, polyphyllin V, polyphyllin VI, polyphyllin VII, polyphyllin B, polyphyllin C, polyphyllin E, polyphyllin F, and polyphyllin H.

Structurally, saponins are glycosides composed of aglycone moieties (sapogenins) linked to sugar chains. Based on their aglycone frameworks, they are classified into steroidal and triterpenoid subtypes (Sparg et al., 2004). As spirostanol-type steroidal saponins, PPS exhibit a characteristic 27-carbon skeleton with sugar moieties attached at the C-3 and/or C-26 positions (Figure 1). Key structural variants include polyphyllin I, II, III, VI, VII, C, and H, among which polyphyllin I, II, VI, and VII are designated by the Chinese Pharmacopoeia as quality-control markers due to their validated anticancer properties (Qin et al., 2018; He et al., 2019).

### 2.1 Bioactive extracts and pharmacological properties

Extracts of Paris polyphylla rhizomes—prepared using solvents ranging from polar (aqueous, alcoholic) to nonpolar (petroleum ether)—demonstrate broad bioactivity. At the extract level, these preparations exhibit antioxidant, antimicrobial, and antitumor effects, which are primarily attributable to their PPS content (Lepcha et al., 2019). Pharmacological studies have systematically characterized PPS as multifunctional agents with anti-inflammatory, analgesic, immunomodulatory (Yang L. et al., 2021), and antitumor activities, along with hemostatic, antimicrobial (Wei et al., 2014), and detoxifying properties (Guan et al., 2019).

### 2.2 Structure-activity relationships and mechanistic insights

The bioactivity of PPS is closely linked to their chemical architecture. For instance, the phenolic hydroxyl groups within saponin structures contribute to antioxidant effects by scavenging free radicals and chelating redox-active metal ions (e.g., Fe^2+^, Cu^2+^), thereby inhibiting lipid peroxidation and hydroxyl radical generation (Li et al., 2021). Notably, the length of sugar chains and the configuration of glycosidic linkages critically influence antimicrobial potency, as exemplified by the stronger activity of polyphyllin I (PPI) compared to polyphyllin H (PPH) (Qin et al., 2012).

### 2.3 Specific bioactive compounds

Among characterized PPS monomers, PPI emerges as a multifunctional candidate. It alleviates oxidative stress via activation of the SIRT3/SOD2/ROS signaling axis, while also demonstrating the highest antimicrobial activity against pathogens such as Cutibacterium acnes and *Staphylococcus aureus* (Firlej et al., 2022). In contrast, PPH exhibits reduced efficacy, highlighting the importance of structural features such as sugar moiety composition for bioactivity (Qin et al., 2012). Furthermore, PPI and related saponins exert antitumor effects through ROS-mediated mechanisms, suppressing proliferation, inducing apoptosis, and reversing multidrug resistance in lung, breast, colorectal, and hepatocellular carcinomas.

## 3 Role of ROS in tumors

ROS primarily originate from mitochondrial oxidative phosphorylation (Cheung and Vousden, 2022; Forman et al., 2014). While regulating cellular homeostasis, ROS induce cytotoxicity via DNA, lipid, and protein damage (Halliwell, 2022; Ismail et al., 2019). This duality drives ROS-mediated tumor promotion and cell death. Cancer cells exploit ROS by dynamically balancing their production, activating oncogenic pathways (e.g., MAPK/NF-κB), and suppressing antioxidants, collectively enhancing tumor progression (Aggarwal et al., 2019).

### 3.1 ROS generation and regulation

**FIGURE 2:**
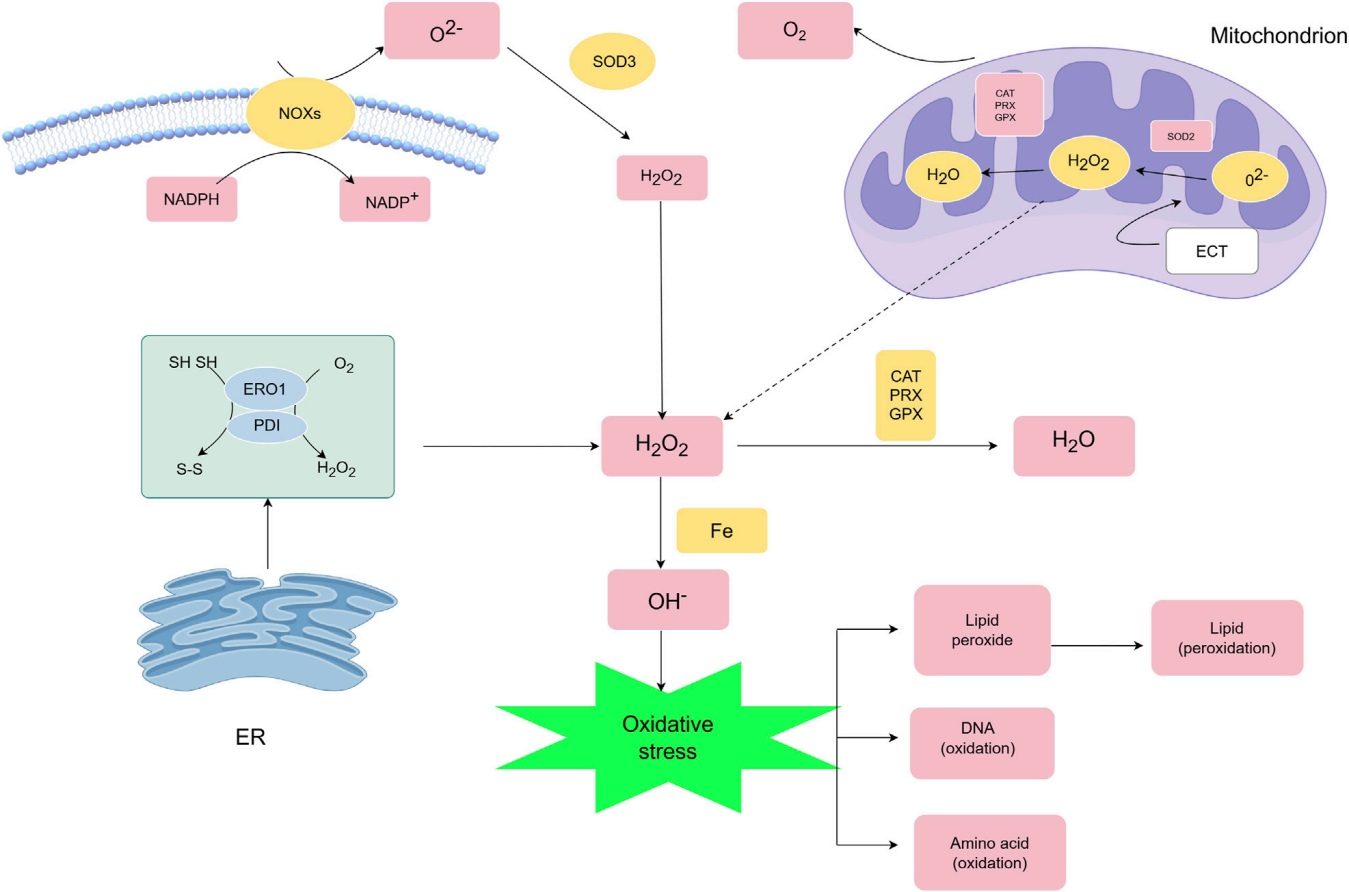
Main generation and modulation of ROS.ROS are primarily generated by mitochondria and cellular membrane NOXs, and their metabolism is regulated by multiple mechanisms. SOD converts O_2_·^-^ into H_2_O_2_. H_2_O_2_ has two metabolic fates: ① it can react with Fe^2+^ through the Fenton reaction to produce hydroxyl radical (OH·), resulting in oxidative injury to cellular macromolecules such as DNA, proteins, and lipids; ② it can be reduced to water by the antioxidant system composed of PRXs, GPXs, and CAT, thereby regulating the intracellular oxidative balance.

Mitochondria represent the predominant intracellular ROS source through electron transport chain (ETC.) activity during oxidative phosphorylation (Figure 2). Approximately 1% of molecular oxygen undergoes partial reduction at complexes I and III, generating superoxide (O_2_^−^) that partitions into the mitochondrial matrix (Complex I) or intermembrane space (Complex III) (Okoye et al., 2023; Murphy, 2009). These radicals are converted to H_2_O_2_ by MnSOD (matrix) and Cu/ZnSOD (cytosol), with cytosolic transfer mediated by mitochondrial permeability transition pores (MPTP).

NADPH oxidases (NOXs) constitute another major ROS-generating system. NOX isoforms (NOX1-5/DUOX1-2) utilize NADPH (maintained by NADK and regulated by MESH1/Nocturnin phosphatases) to produce O_2_^−^ or directly generate H_2_O_2_ (NOX4) through coordinated action of regulatory subunits (Racphox, p47phox, etc.) (Bedard and Krause, 2007; Ding et al., 2020; Schild et al., 2021; Takac et al., 2011).

ROS signaling exhibits spatiotemporal specificity: Physiological H_2_O_2_ diffuses via aquaporins (AQP3/8) to activate redox-sensitive targets, while pathological overproduction disrupts compartmentalization, causing oxidative damage and cell death (Edmondson and Binda, 2018; Pak et al., 2020). Beyond mitochondrial and NOX-derived ROS, secondary sources include endoplasmic reticulum (ER) stress (protein misfolding) (Tu and Weissman, 2002; Haynes et al., 2004), inflammatory cytokines (TNF-α/IL-1β), and hypoxia signaling (Cheung and Vousden, 2022). This dynamic network integrates diverse stimuli to regulate redox homeostasis in health and disease.

### 3.2 The double-edged role of ROS

**FIGURE 3:**
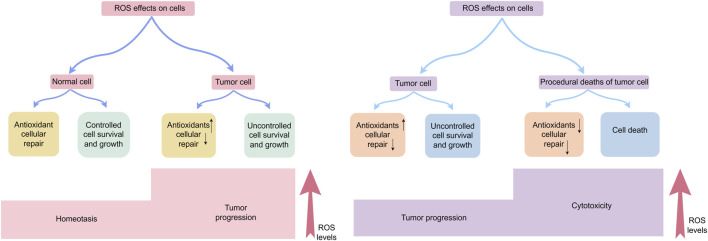
Effects of ROS on cells: physiological function, cancer development, and cell demise.In normal cells, ROS production, antioxidant responses, and cellular repair processes are tightly balanced, maintaining ROS at optimal levels to restrict excessive cell persistence and multiplication. Elevated ROS concentration can induce cellular harm; however, tumor cells often exhibit increased antioxidant capacity and adapt through metabolic reprogramming and hypoxia-induced signaling pathways, supporting tumor-promoting effects. Nevertheless, when ROS surpass a critical threshold, oxidative stress causes irreversible cellular damage, overwhelms adaptive mechanisms, and ultimately triggers tumor cell death.

ROS exhibit concentration-dependent duality in cancer—promoting tumorigenesis at physiological levels through oncogene activation and metabolic reprogramming (Szatrowski and Nathan, 1991), while triggering apoptosis/ferroptosis when exceeding cellular antioxidant thresholds (Wang et al., 2021) This spatiotemporal dynamic positions ROS as both oncogenic drivers and therapeutic targets. Figure 3 delineates their dual roles in homeostasis and tumor progression (Figure 3).

#### 3.2.1 Tumor-promoting effects of ROS

ROS exhibit multifaceted roles in tumorigenesis and cancer progression (Cheung and Vousden, 2022). Chronic oxidative stress induces DNA damage and genomic instability, hallmarks of carcinogenesis (Huang et al., 2022). ROS modulate redox-sensitive signaling pathways in cancer cells through post-translational modifications of cysteine residues in regulatory proteins. These redox-regulated proteins orchestrate pro-tumorigenic processes, including proliferation, metabolic adaptation, invasion, and metastatic dissemination—key drivers of cancer aggressiveness.

##### 3.2.1.1 ROS promotes cell proliferation

ROS play a crucial role in driving cellular survival and proliferation, acting as signaling intermediates in growth factor-mediated pathways, particularly through PI3K/Akt/mTOR and MAPK/ERK pathways, which are central to proliferation and survival (Moloney and Cotter, 2018a). ROS further modulate the nuclear factor kappa B (NF-κB) pathway (Lingappan, 2018) and induce oxidative modifications of transcription factors (e.g., Nrf2, p53), altering their stability and activity in cancer-related processes (Humpton et al., 2022). Collectively, ROS are master regulators of oncogenic signaling networks.

The Akt pathway promotes cell survival by inhibiting pro-apoptotic proteins (e.g., Bad, Bax) and Foxo transcription factors (Essers et al., 2004). ROS-mediated activation of the PI3K/Akt survival pathway represents an early driver of oncogenesis in multiple malignancies. Negative modulators of the PI3K/Akt pathway, including PTEN and PTP1B, contain redox-sensitive cysteine residues in their catalytic sites. Oxidative modification of these residues by H_2_O_2_ inactivates phosphatase activity, resulting in constitutive pathway activation and enhanced tumor cell survival (Hu et al., 2019). This mechanism is observed in various cancer types (Guo et al., 2020).

The MAPK pathway, activated through a three-tiered kinase cascade, regulates proliferation, growth, differentiation, apoptosis, and tumorigenesis. The ERK1/2 branch drives proliferation via growth factor receptors and K-Ras (Khavari and Rinn, 2007). ROS activate ASK1(a member of three-tiered kinase cascade), by oxidizing thioredoxin (TRX), leading to its dissociation from ASK1 (Liu et al., 2000). Beyond upstream regulation, ROS sustain MAPK activation by oxidizing JNK’s cysteine residue to sulfenic acid, preventing dephosphorylation (Son et al., 2011).

ROS can drive drug resistance in tumors. ROS enhance tumor survival via NF-κB/Nrf2 activation, upregulating antioxidant (SOD) and anti-apoptotic (Bcl-2) mediators, directly driving chemoresistance.

##### 3.2.1.2 ROS-driven metastatic progression

ROS drive the process of tumor metastasis: (1) stimulating angiogenesis through *vascular endothelial growth factor(*VEGF) upregulation, and (2) enhancing tumor cell invasion and metastasis via mitochondrial membrane potential (MMP) activation.

ROS drive pathological angiogenesis in cancer through hypoxia-inducible factor-1α (HIF-1α) stabilization, inducing pseudohypoxic HIF complex formation. This activates angiogenic pathways via VEGF upregulation (Catrina and Zheng, 2021; Arbiser et al., 2002; Chatterjee and Chatterjee, 2020; Gerald et al., 2004; Zhao et al., 2023),promoting tumor neovascularization. Beyond angiogenesis, ROS also promote metastasis through integrin/FAK (focal adhesion kinase)-mediated tumor-endothelial adhesion (ten Kate et al., 2006). Paradoxically, targeted suppression of melanoma cells in the microvasculature may elevate endothelial ROS to cytotoxic thresholds, triggering tumor cell apoptosis—a potential antimetastatic strategy (Wang et al., 2000).

**FIGURE 4:**
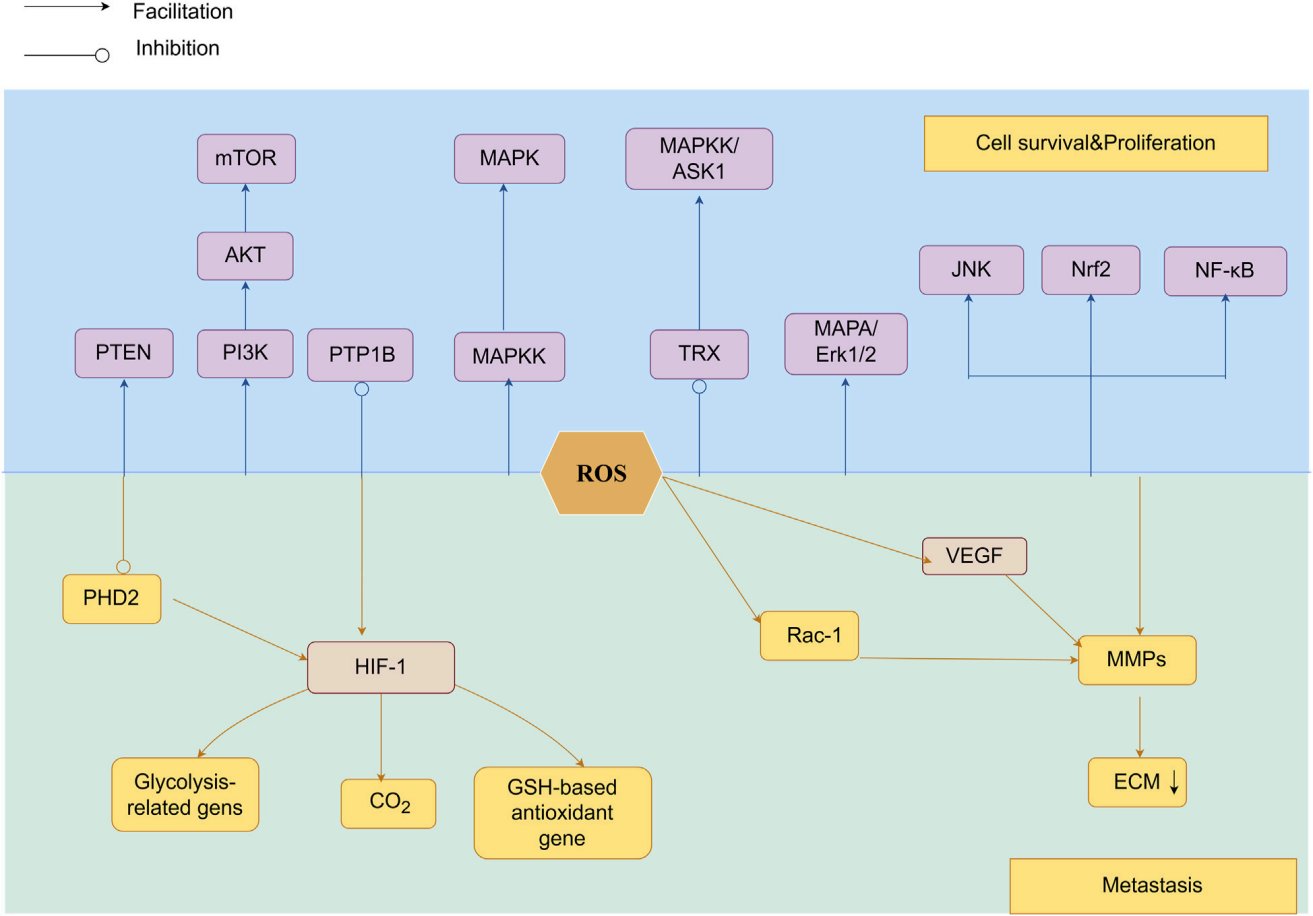
As signaling molecules, ROS participate in the PI3K/Akt/mTOR and MAPK/ERK pathways, modulate NF-κB activity, and are associated with Nrf2 mutations. By inhibiting PHD2 and stabilizing HIF-1α, ROS facilitate tumor cell motility and invasiveness.HIF-1α activation induces the expression of lactate dehydrogenase and pyruvate dehydrogenase kinase 1, suppresses antioxidant genes involved in GSH metabolism, and reduces mitochondrial ROS production, thereby promoting extracellular matrix degradation and invasive behavior. Furthermore, HIF-1α-driven signaling facilitates VEGF-mediated angiogenesis, while ROS accelerate tumor metastasis through MMP-mediated breakdown of ECM proteins, enhancing both vascularization and metastatic spread.

ROS activate matrix metalloproteinases (MMPs) (Nelson and Melendez, 2004)to degrade extracellular matrix (ECM) (Mori et al., 2019), enabling tumor invasion. Aggressive epithelial cancers undergo epithelial-mesenchymal transition (EMT) (Brabletz, 2012), imparting migratory and invasive capacities.ROS-driven EMT enables metastatic colonization. (Wu et al., 2014; Lambert and Weinberg, 2021; Pastushenko et al., 2021).Figure 4.

#### 3.2.2 Carcinogenic effects of ROS

**FIGURE 5:**
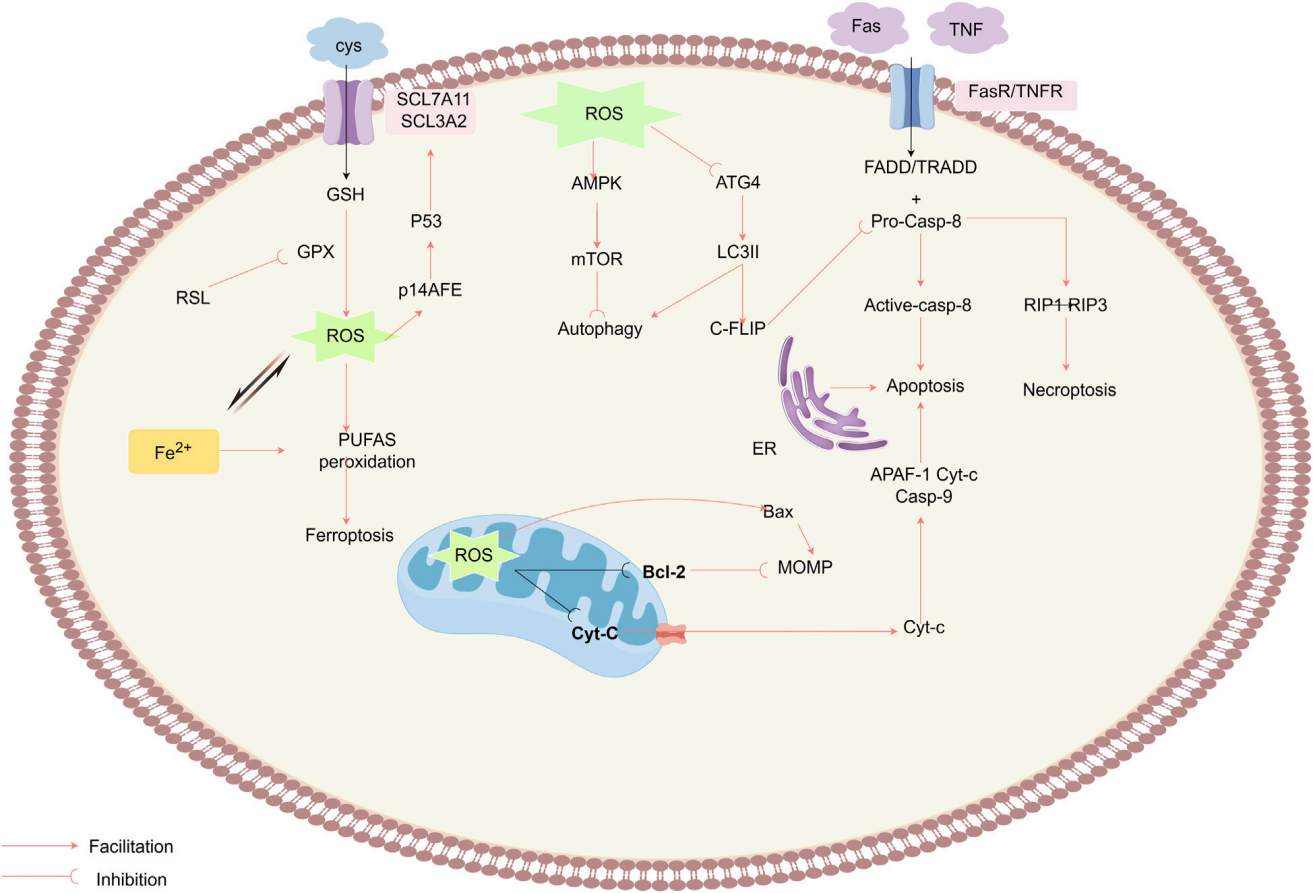
ROS mediate antitumor effects by triggering RCD pathways, including apoptosis, autophagy, necroptosis, and ferroptosis. ROS act on the MPTP, reducing the mitochondrial membrane potential (MMP), which prompts the release of Cyt-c into the cytoplasm, where it binds to APAF-1 and procaspase-9, initiating the caspase-9 cascade reaction and triggering apoptosis.The ROS also enhance the extrinsic apoptosis pathway by degrading c-FLIP and activate RIP1 and RIP3 to induce necroptosis. ROS inactivate the autophagy-related gene Atg4, increase LC3-associated autophagosomes, and promote autophagy, while the inhibition of mTORC1 and the activation of AMPK negatively regulate autophagy. Ferroptosis is an ROS-driven, iron-dependent form of programmed cell death characterized by lipid peroxidation. The Fenton reaction enhances the activity of lipoxygenase and the production of ROS. Erastin disrupts the XC− system, damaging the GPX antioxidant mechanism.Elevated ROS levels disrupt the integrity of the outer mitochondrial membrane, while RSL3 induces ferroptosis by suppressing GPX activity.

ROS accumulation exhibits dual cytotoxic/mutagenic effects, mediating tumorigenesis and cell death. This causes oxidative macromolecular damage (DNA/proteins/lipids) (Bekeschus, 2023), while activating tumor-suppressive programmed cell death (PCD) pathways, including apoptosis, ferroptosis, etc., as intrinsic tumor suppression (Hengartner, 2000). Therapeutically elevating ROS induces tumor-specific PCD (Figure 5) (Liu et al., 2022; Loke et al., 2023).

##### 3.2.2.1 ROS promote apoptosis via extrinsic pathway regulation

Apoptosis (type I PCD) proceeds via extrinsic (death receptor) and intrinsic (mitochondrial) pathways. Extrinsic apoptosis initiates through death receptor (TNFR1/Fas) ligation, recruiting adaptor proteins (e.g., FADD, TRADD) and procaspase-8 to form the death-inducing signaling complex (DISC) (Pan et al., 2022). Procaspase-8 undergoes autocatalytic activation within the DISC, initiating a caspase cascade that executes apoptosis (Wang et al., 2008), counteracted by cellular FLICE inhibitory protein (c-FLIP) through competitive DISC binding. DISC contains death domains (DD/DED), adaptor proteins (FADD/TRADD), and procaspase-8 (Medina et al., 2020). ROS promote this pathway via c-FLIP degradation, enhancing caspase-8 activation (Wilkie-Granth et al., 2013).

The mitochondrial pathway represents the primary ROS-mediated apoptotic route, regulated through MPTPs. ROS oxidize within MPTP components—*voltage-dependent anion channel (VDAC)*, *adenine nucleotide translocase (ANT)*, and *cyclophilin D (CypD)*—increasing membrane permeability (Madesh and Hajnóczky, 2001). This leads to ΔΨm collapse and cytochrome c release (Halliwell, 2022), triggering apoptosome-mediated caspase activation (Bock and Tait, 2020; Kagan et al., 2005; Moloney and Cotter, 2018b; Zuo et al., 2009; Stennicke et al., 1999).

Intrinsic apoptosis is regulated by Bcl-2 family proteins. ROS induce Bcl-2 degradation via ubiquitination-proteasome pathways and upregulate Bax/Bad (Kalkavan et al., 2022; Li et al., 2004). Additionally, ROS activate JNK/p38/MAPK pathways to promote apoptosis (Cadenas, 2004; Liou and Storz, 2010), a mechanism exploited by targeted anticancer therapies (Dimitrov and Marks, 2009). For instance, imatinib triggers ROS-dependent apoptosis in glioblastoma through JNK activation and mitochondrial membrane potential (ΔΨm) collapse (Liu et al., 2019). Similarly, rituximab elevates ROS levels, suppresses Bcl-2, and inhibits p38 MAPK to induce apoptosis in B-cell lymphoma (Alas et al., 2002).

Beyond the extrinsic and intrinsic pathways, ROS modulate apoptosis via the ER stress pathway, which is mediated by cysteine-dependent proteases such as caspase-12. ER stress induced by ROS activates caspase-12 through unfolded protein response (UPR), triggering a caspase cascade that ultimately executes apoptotic cell death (Oppenheim et al., 2001).

##### 3.2.2.2 ROS modulate oncogenesis through autophagy regulation

ROS exhibit dual roles in autophagy, either suppressing or promoting tumorigenesis based on stress intensity (Scherz-Shouval and Elazar, 2011). Autophagy (type II programmed cell death) acts as a cytoprotective mechanism under mild stress but triggers cell death under persistent damage (Debnath et al., 2023; He et al., 2009). Three canonical autophagy forms are recognized: macroautophagy, selective autophagy (e.g., mitophagy), and the microautophagy (Rake et al., 2022). Among its subtypes, selective autophagy (e.g., mitophagy) recruits receptors like p62/SQSTM1 to degrade DAMPs(damage-associated molecular patterns)-marked organelles (Kuchitsu and Taguchi, 2024; Wang et al., 2023). ROS enhance autophagosome accumulation by inhibiting ATG4B-mediated LC3-II delipidation (Perillo et al., 2020). ROS induce autosis through synergism with agents such as 2-mercaptoethanol (2-ME) (Chen et al., 2008). Mechanistically,ROS activate AMPK to suppress mTORC1 (43), thereby initiating ULK1/2-dependent autophagosome formation (Alexander et al., 2010; Poillet-Perez et al., 2015). For instance, H_2_O_2_ combined with the polycyclic ammonium ion sanguinarine amplifies mitochondrial ROS via NOX to induce glioma cell autophagic death (Li et al., 2012). Furthermore, co-targeting mTORC1 (e.g., rapamycin) and HSP90 exacerbates mitochondrial dysfunction, augmenting ROS-driven autophagy against RAS-driven tumors (De Raedt et al., 2011).

##### 3.2.2.3 ROS modulate oncogenesis through ferroptosis regulation

Ferroptosis, an iron-dependent regulated cell death driven by ROS-induced lipid peroxidation (Dixon et al., 2012). This process selectively oxidizes membrane polyunsaturated fatty acids (PUFAs). Its progression depends on iron overload and glutathione (GSH) depletion (Jiang et al., 2015; Jiang et al., 2021; Tang et al., 2021). PUFA oxidation compromises membrane integrity through bilayer destabilization (Dixon and Stockwell, 2019). Cysteine/glutamate anti-transporter - system Xc- and GPX4 - glutathione peroxidase four constitute the major ferrocytic defence axis (Kuang et al., 2020; Liao et al., 2024; Liu K. et al., 2023; Wang et al., 2024).System Xc− imports cystine for GSH synthesis, while GPX4 detoxifies lipid peroxides. Pharmacological inhibitors (e.g., erastin, RSL3) targeting this axis induce ferroptosis (Jang et al., 2021; Kim et al., 2020).Parallel ferroptosis-suppressing pathways include:1)FSP1-CoQH_2_ axis: FSP1(ferroptosis suppressor protein 1)regenerates reduced coenzyme Q10 (CoQH_2_), mitigating lipid radical accumulation. 2)DHODH-CoQH_2_ system: Mitochondrial dihydroorotate dehydrogenase (DHODH) sustains CoQH_2_ pools independent of GPX4. 3)GCH1-BH4 pathway: GTP cyclohydrolase 1 (GCH1) synthesizes tetrahydrobiopterin (BH4), protecting phospholipids from peroxidation (Mao et al., 2021; Mishima et al., 2022; Soula et al., 2020).

Iron serves as a catalytic redox center in ferroptosis by driving lipid peroxidation (Galy et al., 2024). Labile iron pools amplify ROS via Fenton reactions and lipoxygenase (LOX) activation, sustaining a pro-ferroptotic cycle (Conrad and Pratt, 2020; Mishchenko et al., 2021; Yan et al., 2021). Ferroptosis-inducing agents (FIAs), such as erastin, selectively target tumors with iron dysregulation by inhibiting system Xc−, depleting GSH, and elevating mitochondrial ROS (Dolma et al., 2003; Yagoda et al., 2007). While ROS-mediated ferroptosis represents a promising anticancer strategy, emerging evidence cautions its context-dependent effects—e.g., ferroptosis induction combined with macrophage infiltration may paradoxically accelerate pancreatic cancer progression (Dai et al., 2020).

## 4 Anti-tumor mechanism of PPS through regulation of ROS

### 4.1 PPS-induced apoptosis via ROS modulation

Apoptosis, a tightly regulated form of programmed cell death, serves as an important mechanism for maintaining tissue stationary and eliminating malignant cells. PPS demonstrate potent pro-apoptotic activity across multiple cancer cell lineages, thereby validating their therapeutic potential as natural anticancer agents. PPS orchestrate apoptotic signaling via dual regulatory mechanisms: modulation of intracellular ROS homeostasis through both intrinsic (mitochondrial) and extrinsic (death receptor-mediated) pathways.

#### 4.1.1 Mitochondrial activation pathway

The mitochondrial pathway is a primary mechanism through which PPS induce apoptosis. Polyphyllin II (PPII), a major bioactive component of PPS, activates the mitochondrial apoptotic pathway by elevating ROS levels, triggering cytochrome c discharge, and initiating caspase cascade initiation. For instance, PPII suppresses proliferation and induces apoptosis in human gastric cancer MGC-830 cells via caspase-3 activation, mediated by ROS-dependent cytochrome c upregulation. Experimental evidence indicates that polyphyllin VII (PPVII) treatment in bladder cancer cells markedly elevates intracellular and mitochondrial ROS levels, directly linking oxidative stress to mitochondrial apoptosis (Guo et al., 2018).

Increased ROS accumulation reduce mitochondrial membrane potential (ΔΨm), thereby promoting cytochrome c release from mitochondria into the cytosol. This cytosolic cytochrome c activates caspase-9, which subsequently cleaves and activates executioner caspase-3, culminating in apoptosis (Kshetrimayum et al., 2023). Furthermore, PPS activates ROS-generating signaling pathways, including the MAPK and PTEN/p53 axes. These pathways drive ROS accumulation beyond cellular antioxidant capacity, initiating apoptosis through oxidative stress overload. In a cellular model of human hepatocellular carcinoma (HepG2 cells), Zhang et al. systematically evaluated the anti-tumor effects of PPS. After PPVII treatment, JC-1 staining revealed a dose-dependent increase in green/red fluorescence ratio, indicating that PPVII increased ΔΨm collapse and mitochondrial permeability transition pore opening. Concurrently, DCFH-DA fluorescence intensity surged, confirming PPVII-induced ROS overproduction. Western blot analysis demonstrated significant phosphorylation enhancement of MAPK pathway components—JNK, ERK, and p38. These findings mechanistically link PPVII-induced mitochondrial dysfunction and ROS overgeneration to MAPK/PTEN-p53 pathway activation, establishing a cascading pro-apoptotic signaling network (Zhang et al., 2016).

#### 4.1.2 Activation of death receptor pathways

PPS additionally modulate intracellular ROS to activate extrinsic apoptotic pathways in cancer cells. PPS upregulates death receptor expression (e.g., Fas/CD95, TNFR) on tumor cell surfaces, initiating extrinsic apoptosis (Hengartner, 2001). Liu and his teams demonstrated that Paris polyphylla saponin VI (PPVI) suppresses HepaRG cells viability through Fas-dependent apoptosis. Western blot analysis revealed PPVI-induced upregulation of Fas, caspases-3/8/9, and Poly (ADP-ribose) polymerase (PARP) cleavage, confirming activation of the extrinsic pathway. PPVI treatment elevated intracellular ROS levels, inducing oxidative stress that triggered Fas overexpression and caspase-8 activation. Caspase-8 subsequently activated executioner caspase-3, culminating in apoptosis.These findings indicate that PPVI triggers apoptosis in HepaRG cells via the Fas-dependent extrinsic pathway (Liu et al., 2018). Similarly, PPI dose and time-dependently elevated ROS levels, disrupted mitochondrial membrane potential (ΔΨm), and promoted cytochrome c release in HepG2 cells. Concurrently, PPI upregulated Fas, p53, and p21 expression, increased Bax/Bcl-2 ratios, and activated caspases-3/8/9, leading to PARP cleavage and apoptosis (Zeng et al., 2020). Lin et al. have provided additional evidence that resorcinolic acid saponins activate the extrinsic apoptotic pathway to induce cancer cell death in an A549 xenograft model. Treatment with saponin fractions PPVI and PPVII elevated intracellular ROS concentrations, thereby initiating oxidative stress and upregulating death receptor expression. Western blot analysis of both cultured cells and xenograft tumor tissues revealed that PPVI/PPVII treatment upregulated pro-apoptotic regulators (p53, p21WAF1/CIP1), death receptors (DR3, DR5, Fas), and apoptosis execution markers (cleaved PARP, caspase-3), while downregulating cell cycle promoters cyclin B1 and decoy receptor DcR3. These death receptors (DR3, DR5, Fas) initiate proteolytic cascades that activate executioner caspases (-3, -8, -9), mechanistically linking TRAIL- and Fas-mediated apoptosis pathways (Lin et al., 2015).Furthermore, Ke et al. validated the pro-apoptotic effects of PPS in tongue squamous cell carcinoma, demonstrating ROS-dependent activation of the caspase-8/caspase-3 axis as the principal mechanism. As the initiator caspase in extrinsic apoptosis, caspase-8 activation represents a critical regulatory node governing programmed cell death. Through formation of the death-inducing signaling complex (DISC), caspase-8 catalyzes proteolytic activation of downstream effectors, committing cells to apoptosis (Ke et al., 2016).

#### 4.1.3 Endoplasmic reticulum stress and Other pathways

ER stress-induced apoptosis is a key mechanism by which PPS exert their effects on oxidative stress regulation. Previous studies have shown that polyphylla saponins induce apoptosis in lung cancer cells through modulation of ER stress, resulting in significantly increased intracellular oxidative stress and ROS accumulation. Researchers conducting cDNA microarray analysis on polyphylla saponin-treated lung cancer cells observed significant upregulation of multiple ER stress-associated genes. Western blot analysis confirmed the elevated expression of ER stress markers including BiP/GRP78, PDI, and HSP70, demonstrating ER stress activation and its association with apoptosis induction (Siu et al., 2008). In a seminal study, Tan’s team established the inhibitory effects of polyphylla saponin on nasopharyngeal carcinoma cells using *in vitro* models. Quantitative Western blot analysis revealed both enhanced PERK phosphorylation and upregulated expression of ER stress mediators including CHOP, BiP, PDI, ERO1α, and IRE1α, thereby confirming ER stress activation. Consistent with the established pro-apoptotic role of ER stress, morphological analysis revealed characteristic apoptotic features in CNE-2 cells post-treatment, including nuclear fragmentation, chromatin condensation, and cytoplasmic shrinkage. Flow cytometry with Annexin V-FITC/PI staining quantitatively demonstrated increased apoptotic cell populations, thereby providing multimodal evidence for ER stress-mediated apoptosis (Tan et al., 2019).

### 4.2 PPS promotes cancer cell autophagy by regulating ROS

Autophagy is a conserved intracellular degradation process that maintains cellular homeostasis through lysosomal recycling of superfluous or damaged cytoplasmic components (Miller and Thorburn, 2021). The dual role of autophagy in tumorigenesis—acting as both a pro-survival and tumor-suppressive mechanism—is well characterized. While autophagy may sustain cancer cell survival under metabolic stress by replenishing energy and nutrients, it can also trigger autophagic cell death or suppress tumor progression under specific contexts (Gozuacik and Kimchi, 2004). Emerging evidence highlights ROS as critical regulators of autophagy (Chen et al., 2009). ROS accumulation activates AMPK, a master energy sensor that induces autophagy under low energy conditions (e.g., ATP depletion), thereby restoring metabolic homeostasis in cancer cells (Carling, 2017).

**FIGURE 6:**
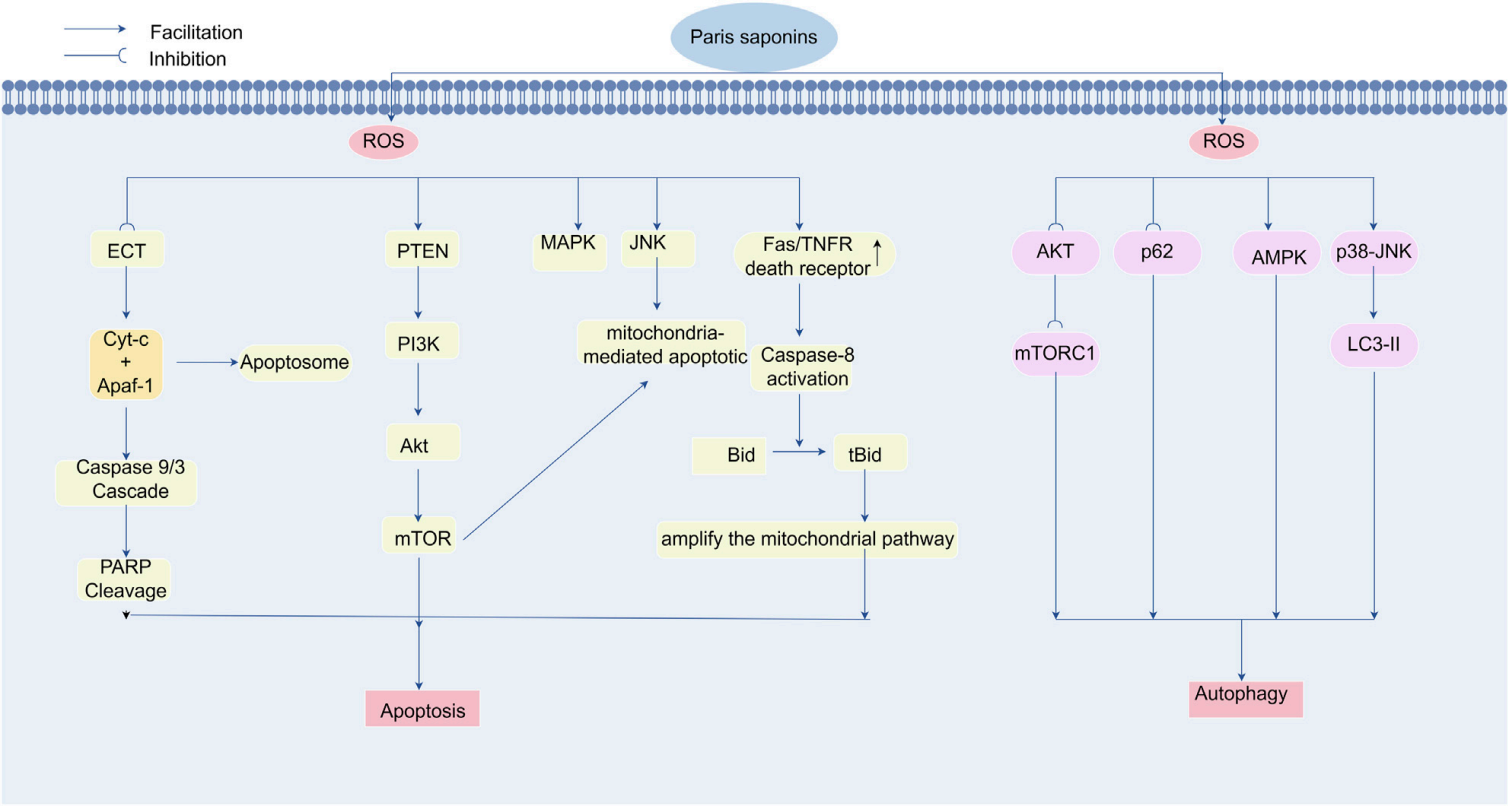
Polyphyllin triggers apoptosis and autophagy for cancer treatment by regulating reactive oxygen species levels. Paradisaponin increases intracellular ROS levels, triggering oxidative stress, which in turn activates endogenous and exogenous apoptosis pathways, leading to the death of cancer cells. It can elevate ROS levels in the mitochondria and cytoplasm, resulting in a decrease in mitochondrial membrane potential, the release of cytochrome c, and the subsequent activation of the Caspase cascade system, leading to PARP cleavage and DNA fragmentation, ultimately triggering apoptosis. Paradisaponin, by increasing ROS levels, inhibits the AKT/mTOR pathway, activates the AMPK and MAPK pathways to promote LC3-II expression, and induces autophagy in cancer cells.

Luo et al. demonstrated that PPI induces autophagy in colon cancer cells at specific therapeutic concentrations. This autophagic activity was evidenced by an elevated LC3-II/LC3-I ratio, a hallmark of autophagosome formation. Concurrently, PPI treatment in SW480 cells induced ROS accumulation and suppressed AKT phosphorylation, effects reversible by N-acetylcysteine (NAC) cotreatment. These findings indicate ROS-dependent AKT/mTORC1 pathway inhibition as the mechanistic basis for PPI-mediated autophagy induction (Luo et al., 2022).Similarly, PPVII activates autophagic flux in glioma models through LC3-II upregulation coupled with AKT inactivation and SQSTM1/p62 degradation (Pang et al., 2020). Notably, PPI exhibits dual pro-death effects in temozolomide-resistant gliomas, coordinately activating p38-JNK MAPK signaling to drive ROS-mediated apoptosis and autophagy (Feng et al., 2024).Yuan and his team further confirmed that PPVI triggers autophagy in U2OS osteosarcoma cells through ROS modulation. Mechanistically, PPVI elevates intracellular ROS via H_2_O_2_ generation, activating the JNK signaling pathway. This cascade promotes autophagy as evidenced by LC3-II accumulation, with JNK activation showing direct correlation to autophagic marker upregulation, confirming its pivotal role in PPVI-mediated autophagy (Yuan et al., 2019).Figure 6.

### 4.3 ROS-mediated cell cycle arrest by PPS

Effective suppression of tumorigenesis and progression is contingent upon arresting cancer cell proliferation. Malignant cells evade growth-suppressive checkpoints through oncogenic reprogramming, acquiring limitless replicative potential. Such dysregulated proliferation imposes systemic pathophysiological stress and disrupts tissue homeostasis.The tumor suppressor p53 orchestrates cellular homeostasis through pleiotropic regulation of growth arrest, DNA repair, and apoptotic pathways. As a key p53 effector, p21 (CDKN1A) mediates cell cycle arrest by inhibiting CDK2-cyclin complexes and modulating PCNA functionality. p21 overexpression induces G1 phase arrest, effectively suppressing neoplastic growth across experimental models (Gartel et al., 1996). PPS exert antitumor effects through pharmacologically-induced cell cycle arrest (Tian et al., 2020).

Elevated ROS levels modulate cell cycle progression by targeting key regulatory proteins. ROS overproduction upregulates *p21* expression and its downstream effector cyclin B1, a critical driver of G2/M phase transition (Nagesh et al., 2017). Yu et al. demonstrated that PPI induces concentration-dependent G2/M phase arrest, an effect reversible by NAC pretreatment. Notably, PPI selectively modulates cell cycle regulators: downregulating cyclin B1 while upregulating *p21* in a concentration-dependent manner, without altering CDC2, p27, CDC25C, or GAPDH expression. These findings delineate a ROS-dependent mechanism underlying PPI-induced G2/M arrest (Yu et al., 2018). Complementary studies in HepaRG models revealed that PPII triggers ROS accumulation and mitochondrial depolarization, concomitant with S-phase arrest. Antioxidant pretreatment with NAC attenuates these effects, restoring membrane potential and reducing apoptotic commitment (Wang et al., 2019) Figure 7.

**FIGURE 7:**
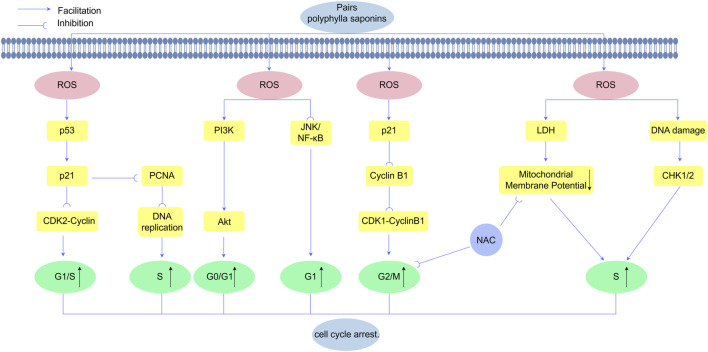
Polyphyllin exerts anticancer effects by inducing ROS-mediated cell cycle arrest in cancer cells. Polyphyllin increases ROS levels to activate the PI3K/Akt signaling cascade and JNK/NF-κB, leading to cell cycle arrest at the G0/G1 and G1 phases The upregulation of p53 promotes the expression of p21, which inhibits CDK2-cyclin complexes and proliferating cell nuclear antigen (PCNA), thereby blocking G1/S phase transition and inducing S phase arrest. Additionally, Polyphyllinsuppresses cyclin B1 expression, disrupting the CDK1-cyclin B1 complex and resulting in G2/M phase arrest.

### 4.4 PPS promote ferroptosis in cancer cells by modulating ROS

Ferroptosis is a regulated cell death mechanism that relies on iron and is marked by lipid peroxidation and excessive intracellular iron accumulation. Excessive iron accumulation and GSH depletion synergistically drive oxidative stress, resulting in lethal peroxidation of PUFAs. PPS induce ferroptosis in cancer cells through ROS-mediated mechanisms. For instance, PPI suppresses acute myeloid leukemia progression by activating the PI3K/SREBP-1/SCD1 axis to promote lipid oxidative damage and ferroptosis (Zhou et al., 2024).Yang and his team demonstrated that PPI induces ferroptosis in hepatocellular carcinoma (HCC) cells with elevating intracellular Fe^2+^ and ROS levels, depleting GSH, increasing malondialdehyde (MDA), and downregulating xCT and GPX4 expression. In a nude mouse xenograft model, PPI significantly suppressed tumor growth and inhibited the Nrf2/HO-1 antioxidant axis. This suppression reduced GPX4 activity, further amplifying ROS accumulation and iron overload, ultimately driving HCC cells into ferroptosis (Yang et al., 2024). Feng et al. demonstrated that PPI suppresses the Nrf2/HO-1 antioxidant pathway in glioblastoma, thereby elevating ROS levels and inducing ferroptosis. These results emphasize the therapeutic potential of PPI for drug-resistant gliomas (Feng et al., 2024). Furthermore, Zou et al. revealed that PPI modulates the ERK/DNMT1/ACSL4 axis in castration-resistant prostate cancer (CRPC) cells, driving ferroptosis through elevated MDA, Fe^2+^, and ROS, alongside reduced GSH and GPX4 levels—a hallmark of ferroptosis (Zou et al., 2024).

### 4.5 PPS in combination with drugs reverses resistance and improves therapeutic efficacy

Clinical applications of ROS-modulating monotherapies frequently demonstrate limited efficacy in tumor growth suppression. However, combinatorial regimens integrating these compounds with conventional chemotherapeutic agents not only potentiate natural product efficacy but also reverse chemoresistance via synergistic ROS-mediated mechanisms. Pang et al. demonstrated that PPVII modulates the Bcl-2/Bax apoptotic rheostat, thereby initiating mitochondrial pathway apoptosis. Mechanistically, PPVII administration significantly suppressed phospho-AKT/mTORC1 signaling axis activity. N-acetylcysteine (NAC) pretreatment abolished PPVII-mediated cytotoxicity, autophagic flux, and AKT/mTORC1 suppression, establishing ROS overproduction as the central mechanistic driver of these phenomena. The PPVII-TMZ combination exhibited marked synergism in glioma models, particularly in overcoming O6-methylguanine-DNA methyltransferase (MGMT)-mediated chemoresistance. This sensitization correlated with PPVII-induced epigenetic silencing of MGMT via promoter hypermethylation (Pang et al., 2019). Chen’s group elucidated that PPVII-paclitaxel coadministration provokes profound ROS burst, culminating in mitochondrial depolarization, ferroptosis induction, and complete reversal of prostate cancer chemoresistance (Chen et al., 2025). Zhang et al. pioneered the discovery that Polyphyllin D (PPD) dose- and time-dependently induces mitochondrial transmembrane potential (ΔΨm) collapse, H2O2 accumulation, and ROS-mediated apoptosis in chemoresistant HepG2 cells, thereby establishing PPD as a first-in-class mitochondrial disruptor capable of bypassing hepatocellular carcinoma chemoresistance (Cheung et al., 2005). Notably, PPS exhibits broad-spectrum chemosensitization potential, effectively reversing resistance to erlotinib (NSCLC), gefitinib (LUAD), and cisplatin (TNBC) while preventing acquired resistance in these malignancies (Lou et al., 2017; Zhang et al., 2024; Al Sawah et al., 2015). Collectively, these findings position PPS as a multimodal chemopotentiator that enhances conventional chemotherapy (paclitaxel, temozolomide, cisplatin) through three-pronged mechanisms: (1) ROS amplification, (2) p53-PUMA-Bax apoptotic axis activation, and (3) mitochondrial death pathway engagement.

### 4.6 Interaction of PPS with other signaling pathways by regulating ROS

Signaling pathways are pivotal in cellular function, serving as the primary mechanisms through which cells perceive external stimuli and coordinate physiological responses. These pathways orchestrate diverse cellular processes, including differentiation, survival, apoptosis, migration, invasion, metabolism, stress adaptation, and cell cycle progression. Through intricate crosstalk within a signaling network, these pathways collectively enable cellular adaptation to environmental perturbations while preserving homeostasis. Beyond the aforementioned pathways, PPS exhibit anti-tumor effects by modulating ROS to target additional signaling cascades (Zhao et al., 2021) Table.1.

**TABLE 1:** The application of PPS in cancer treatment.

Types of cancer	Animals/cell types	Dosage	Theraputic effect	Source
Hepatocellular Carcinoma, HCC	The human hepatocellular carcinoma cell line	Cytotoxicity tested with concentrations ranging from 2 μM to 16 µM for 24 and 48 h Further experiments used concentrations of 2, 4, 6, 8, and 12 µM for 24 h	The study suggests that polyphyllin VI has potent cytotoxic effects on HepaRG cells, primarily through apoptosis mediated by the Fas death pathway and the mitochondrial pathway	Liu et al. (2018)
Colon cancer	SW480 cell line	IC50 values (50% inhibitory concentration) were determined for 12 and 24 h treatment: 4.9 ± 0.1 μmol/L for 12 h and 3.5 ± 0.2 μmol/L for 24 h	PPI markedly suppressed SW480 cell growth in a dose-dependent fashion	Luo et al. (2022)
Lung cancer	Nude mice xenograft model with A549 cells	PVI: 2 mg/kg, 3 mg/kg, 4 mg/kg PVII: 1 mg/kg, 2 mg/kg, 3 mg/kg	*In vitro*:PVI and PVII suppress the proliferation of A549 and NCI-H1299 cells, trigger G2/M phase cell cycle arrest, and promote apoptosis *In vivo*: PVI and PVII can significantly inhibit the growth of xenograft tumors in nude mice without affecting their body weight	Lin et al. (2015)
Human osteosarcoma	U2OS cell line	CCK8 assay: 0, 1, 2, 4, 6, 8, 12, and 30 μM Apoptosis and autophagy experiments: 0, 2.5, 5, and 7.5 μM ROS and JNK experiments: 0, 2.5, 5, and 7.5 μM H2O2 experiments: 0, 2.5, 5, and 7.5 μM	PPVI effectively triggers apoptosis and autophagy in osteosarcoma cells through activating the ROS/JNK signaling pathway, thereby inhibiting cell growth and showing potential for the treatment of osteosarcoma	Yuan et al. (2019)
Hepatocellular Carcinoma, HCC	Nude mice xenograft model (MHCC97H cells)	*In vitro*: 2, 4, 6, 10, 20 μM *In vivo*: 1.5 mg/kg, 3 mg/kg (intraperitoneal injection daily)	PPI induces ferroptosis in HCC cells and causes mitochondrial structural and functional damage by modulating the Nrf2/HO-1/GPX4 axis, thereby inhibiting the proliferation, migration, and invasion of HCC cells and suppressing HCC tumor growth	Yang et al. (2024)
Hepatocellular carcinoma (HCC)	human liver cancer cell lines	PPVII was tested at various concentrations, with the IC50 value for HepG2 cells at 1.32 ± 0.04 μM for 24 h	PPVII demonstrated strong anticancer activity against HepG2 cells through multiple mechanisms, including apoptosis induction, mitochondrial dysfunction, ROS production, and activation of PTEN/p53 and MAPK signaling pathways	Zhang et al. (2016)

## 5 Other bioactivities of PPS

The NF-κB signaling pathway is a central transcriptional regulator of inflammatory responses. Macrophages activate NF-κB through Toll-like receptors (TLRs), triggering the secretion of pro-inflammatory factors, including NO, prostaglandin E2 (PGE2), COX-2, MMPs, TNF-α, IL-1β, and IL-6. These mediators attract immune cells to sites of infectious sites or injured tissue (Mussbacher et al., 2019; Moncada, 1999). In canonical NF-κB activation, stimuli like lipopolysaccharide (LPS), interferon-γ (IFN-γ), and TNF-α induce IκB kinase (IKK) complex activation. IKK phosphorylates specific serine residues (e.g., Ser32/36) on IκBα, marking it for proteasomal degradation. IκBα degradation liberates NF-κB, enabling its nuclear translocation and subsequent transcription of pro-inflammatory genes (e.g., TNF-α, IL-1β, IL-6, iNOS) (Fearon et al., 2016).

PPS exert multi-faceted anti-inflammatory effects by targeting key nodes in this pathway.PPS exhibit dose-dependent anti-inflammatory activity by suppressing key pro-inflammatory mediators, including TNF-α (Zhu et al., 2019), IL-1β (Yang S. et al., 2021), NO, IL-1α, IL-6, and IL-8. In collagen-induced arthritis (CIA) murine models, PPI alleviates joint inflammation by inhibiting NF-κB-dependent inflammatory cytokine production in macrophages. These findings highlight the therapeutic potential of PPI for rheumatoid arthritis (RA) management (Wang et al., 2018). PPVII suppresses LPS-triggered macrophage activation primarily through NF-κB pathway inhibition. LPS stimulation induces IκB-α phosphorylation and degradation, enabling NF-κB p65 nuclear translocation and subsequent transcription of pro-inflammatory genes. PPVII attenuates this cascade by stabilizing IκB-α and blocking NF-κB p65 nuclear translocation, thereby reducing pro-inflammatory mediator synthesis. Furthermore, PPVII inhibits LPS-activated JNK, ERK, and p38 MAPK signaling, suggesting an additional mechanism to suppress IκB-α degradation (Zhang et al., 2019).

In addition to the above inflammation-related diseases, PPS can also play an anti-tumor role by regulating inflammatory factors, Chen et al. elucidated that PPII suppresses NF-κB activation via blockade of IKKβ/p65 nuclear translocation, thereby suppressing colorectal cancer progression (Chen et al., 2019). Studies have demonstrated PPI concentration-dependently enhances HepG2 cell chemosensitivity to cisplatin.Mechanistically, PPI dose-dependently attenuates basal phosphorylation of the NF-κB p65 subunit and downregulates downstream oncogenic targets (Bcl-2, c-Myc, VEGF), priming HepG2 cells for chemotherapy response (Han et al., 2015). In translational models, PPI potently suppressed CRPC growth and induced G1/S phase arrest in both xenograft models and primary cell cultures.PPI concomitantly reduced p65 phosphorylation, mucin 1 (MUC1) oncoprotein levels, and long non-coding RNA HOTAIR expression.These findings establish PPI as multimodal inhibitors that disrupt NF-κB/p65-MUC1 signaling crosstalk in stroma-rich prostate tumors (Xiang et al., 2018).

In summary, inflammation and cancer exhibit bidirectional crosstalk, forming a self-amplifying pathological cycle. Tumor cells perpetuate inflammation through pro-inflammatory cytokine secretion, whereas chronic inflammatory microenvironments promote oncogenesis, tumor progression, and therapeutic resistance. As a dual anti-inflammatory and anti-neoplastic agent, PPS suppress NF-κB signaling and attenuate inflammatory cytokine activity, thereby disrupting the inflammation-cancer axis and suppressing tumor progression.

## 6 Conclusion and outlook

In conclusion, preclinical studies of PPS in cellular and animal models demonstrate promising antitumor efficacy; however, clinical translation remains limited by critical challenges: 1) Low bioavailability: The poor aqueous solubility of PPS limits gastrointestinal absorption, resulting in suboptimal systemic exposure and heterogeneous tissue distribution. 2) Rapid hepatic metabolism: PPS undergoes extensive first-pass metabolism, generating inactive or potentially toxic metabolites that compromise therapeutic efficacy. 3) Drug-drug interactions: PPS may interfere with cytochrome P450 enzymes, altering the pharmacokinetics of co-administered therapeutics. 4) Dose-limiting toxicity: While PPS exhibits potent *in vitro* antitumor activity, *in vivo* studies report hepatotoxicity, nephrotoxicity, and myelosuppression at elevated doses, necessitating rigorous safety profiling. 5) Insufficient translational data: Gaps in pharmacokinetic-pharmacodynamic (PK/PD) modeling and early-phase clinical trials hinder rational dose optimization and risk-benefit assessment.

In future research, the following strategies should be prioritized to address these limitations: 1) Pharmacokinetic optimization: Advanced nano-delivery platforms (e.g., liposomes, exosomes) require systematic development to overcome the poor aqueous solubility and limited bioavailability of PPS. 2) Toxicity mitigation: Structural engineering approaches, such as site-specific glycosylation or prodrug design, should be investigated to alleviate dose-dependent hepatotoxicity while preserving bioactivity. 3) Clinical translation: Phase I/II clinical trials must be initiated to establish safety profiles, determine optimal dosing regimens, and validate therapeutic efficacy in human populations, building upon current preclinical evidence. 4) Combinatorial therapy exploration: Synergistic regimens integrating PPS with immune checkpoint inhibitors or targeted therapies could enhance therapeutic indices while minimizing systemic toxicity. 5) Biomarker-driven stratification: Identification of predictive biomarkers (e.g., Nrf2 expression, ROS levels) may enable personalized dosing and patient selection.
